# Prediction of Myocardial Infarction Using a Combined Generative Adversarial Network Model and Feature-Enhanced Loss Function

**DOI:** 10.3390/metabo14050258

**Published:** 2024-04-30

**Authors:** Shixiang Yu, Siyu Han, Mengya Shi, Makoto Harada, Jianhong Ge, Xuening Li, Xiang Cai, Margit Heier, Gabi Karstenmüller, Karsten Suhre, Christian Gieger, Wolfgang Koenig, Wolfgang Rathmann, Annette Peters, Rui Wang-Sattler

**Affiliations:** 1 TUM School of Medicine and Health, Technical University of Munich, 81675 München, Germany; shixiang.yu@helmholtz-munich.de (S.Y.); siyu.han@helmholtz-munich.de (S.H.); mengya.shi@helmholtz-munich.de (M.S.); jianhong.ge@helmholtz-munich.de (J.G.); 2 Institute of Translational Genomics, Helmholtz Zentrum München, German Research Center for Environmental Health, 85764 Neuherberg, Germany; makoto.harada@helmholtz-munich.de; 3 German Center for Diabetes Research (DZD), 85764 Neuherberg, Germany; 4 Biocomputing R&D Department, Beijing Huanyang Bole Consulting Co., Ltd., Beijing 100010, China; ning@phdboler.com; 5 School of Computer Science and Information Security, Guilin University of Electronic Technology, Guilin 541214, China; xiangcai@keter.top; 6 KORA Study Centre, University Hospital of Augsburg, 86153 Augsburg, Germany; margit.heier@helmholtz-munich.de; 7 Institute of Epidemiology, Helmholtz Zentrum München, German Research Center for Environmental Health, 85764 Neuherberg, Germany; annette.peters@helmholtz-munich.de; 8 Institute of Computational Biology, Helmholtz Zentrum München, German Research Center for Environmental Health, 85764 Neuherberg, Germany; gabi.kastenmuller@helmholtz-munich.de; 9 Department of Physiology and Biophysics, Weill Cornell Medicine and Director of the Bioinformatics Core, Doha 24144, Qatar; karsten@suhre.fr; 10 Research Unit of Molecular Epidemiology, Helmholtz Zentrum München, German Research Center for Environmental Health, 85764 Neuherberg, Germany; christian.gieger@helmholtz-munich.de; 11 Deutsches Herzzentrum München, Technische Universität München, 80636 München, Germany; koenig@dhm.mhn.de; 12 Institute for Biometrics and Epidemiology, German Diabetes Center, Leibniz Center for Diabetes Research, Heinrich Heine University, 40225 Düsseldorf, Germany; rathmann@ddz.de; 13 Institute for Medical Information Processing, Biometry, and Epidemiology (IBE), Pettenkofer School of Public Health, Faculty of Medicine, Ludwig-Maximilians-Universität München, 81377 München, Germany

**Keywords:** myocardial infarction, prediction, generative adversarial networks, limited and imbalanced incident cases, feature enhancement, GAN for feature-enhanced, GFE loss function

## Abstract

Accurate risk prediction for myocardial infarction (MI) is crucial for preventive strategies, given its significant impact on global mortality and morbidity. Here, we propose a novel deep-learning approach to enhance the prediction of incident MI cases by incorporating metabolomics alongside clinical risk factors. We utilized data from the KORA cohort, including the baseline S4 and follow-up F4 studies, consisting of 1454 participants without prior history of MI. The dataset comprised 19 clinical variables and 363 metabolites. Due to the imbalanced nature of the dataset (78 observed MI cases and 1376 non-MI individuals), we employed a generative adversarial network (GAN) model to generate new incident cases, augmenting the dataset and improving feature representation. To predict MI, we further utilized multi-layer perceptron (MLP) models in conjunction with the synthetic minority oversampling technique (SMOTE) and edited nearest neighbor (ENN) methods to address overfitting and underfitting issues, particularly when dealing with imbalanced datasets. To enhance prediction accuracy, we propose a novel GAN for feature-enhanced (GFE) loss function. The GFE loss function resulted in an approximate 2% improvement in prediction accuracy, yielding a final accuracy of 70%. Furthermore, we evaluated the contribution of each clinical variable and metabolite to the predictive model and identified the 10 most significant variables, including glucose tolerance, sex, and physical activity. This is the first study to construct a deep-learning approach for producing 7-year MI predictions using the newly proposed loss function. Our findings demonstrate the promising potential of our technique in identifying novel biomarkers for MI prediction.

## 1. Introduction

Myocardial infarction (MI) is a significant global health concern and remains a leading cause of death worldwide [1]. Accurate prediction of MI risk is crucial for early intervention and prevention of the disease [2,3]. Metabolomics has emerged as an innovative approach for identifying potential biomarkers and risk factors associated with MI [1,4,5,6,7]. However, the availability of population-based human cohorts with comprehensive metabolite profiles for incident MI cases is often limited and imbalanced, with a scarcity of non-MI individuals [6]. For example, in a study by Nogal et al., a two-step meta-analysis was conducted using data from the COnsortium of METabolomics Studies, involving 7897 individuals, including 1373 incident MI cases from six cohorts [6]. Alternatively, nested study designs have been employed, matching cases with non-cases in a 1:1 ratio [5]. However, the selection of non-MI cases in population-based studies may introduce bias and potentially lead to disparate results [8]. Furthermore, the limited and imbalanced number of incident cases poses challenges when applying machine learning (ML) methods for prediction. Therefore, the development of a novel MI prediction model is warranted.

In recent years, ML techniques, such as support vector machine (SVM) [9] and random forest (RF) [10], have been extensively utilized to forecast disease risk due to their improved accuracy and sensitivity, particularly with complex data. SVM and RF algorithms, for instance, have been extensively employed to identify biomarkers associated with chronic renal disease [11] and to detect MI based on electrocardiographic (ECG) signals [12,13,14]. Additionally, decision tree (DT) and RF models have been utilized to identify key microbial species linked to colorectal cancer [15]. Moreover, k-nearest neighbor (k-NN) algorithms have been employed to identify significant indicators of breast cancer based on anthropometric and clinical characteristics [16]. These studies demonstrate the effectiveness of ML as a tool for disease diagnosis and biomarker discovery.

The current ML algorithms often treat clinical variables and metabolite profiles as separate entities, failing to capture the potential associations between these datasets, particularly when dealing with vast amounts of omics data. Deep-learning (DL) algorithms, such as the multilayer perceptron (MLP), offer a promising solution in this regard [17,18,19,20,21]. In a previous study, an MLP-based approach was employed to predict heart disease, including MI, using the Cleveland Heart Disease dataset. However, the performance of DL models can be hampered by limited datasets.

To address this limitation, a DL method known as generative adversarial network (GAN) has emerged [22,23,24,25,26]. GAN has the capability to generate new data and transfer styles by leveraging the mappings of the original data [27]. This unique characteristic empowers the GAN model to enhance features and expand the potential distribution of limited data at a relatively low cost [28].

In this study, our primary objectives are threefold. Firstly, we aim to develop a novel DL method that utilizes a GAN model for feature enhancement, enabling the establishment of a 7-year MI prediction model. This DL approach will be built upon real observational metabolomic and phenotypic data obtained from the KORA (Cooperative Health Research in the Region of Augsburg) human cohort.

Secondly, we propose a novel loss function, GAN for feature-enhanced (GFE), designed to further enhance the prediction accuracy of our DL model. By incorporating this unique loss function, we aim to improve the model’s ability to identify subtle yet critical patterns and associations within the data, leading to more robust and accurate MI risk predictions.

Lastly, we seek to interpret the DL model and leverage its capabilities to identify metabolites and clinical variables that are strongly correlated with MI. By unraveling these associations, we aim to gain valuable insights into the underlying biological mechanisms and potential biomarkers of MI, ultimately contributing to a better understanding of the disease and its predictive indicators.

## 2. Methods

### 2.1. KORA Cohort

The KORA cohort is a population-based study conducted in the city of Augsburg and the surrounding towns and villages in southern Germany [29]. For this study, we utilized data from the baseline KORA survey 4 (KORA S4), which was conducted between 1999 and 2001 and included 4261 participants aged between 25 and 74 years [30]. Additionally, a follow-up survey (KORA F4) was conducted from 2006 to 2008, with 3080 participants taking part [31,32].

### 2.2. MI Definition

Myocardial infarction (MI) is clinically defined as the death of cardiac muscle tissue caused by prolonged ischemia. Ischemia occurs when there is a blockage or reduced blood flow to a part of the heart, leading to a lack of oxygen supply [33].

In our study, only non-fatal MI events were included. Both fatal and non-fatal MI events were identified through two primary sources: the KORA Augsburg coronary event registry and participant questionnaires for individuals residing outside the study area [1]. Until 31 December 2000, the diagnosis of MI events was based on the World Health Organization (WHO) Multinational Monitoring of Trends and Determinants in Cardiovascular Diseases (MONICA) algorithm, which took into account symptoms, cardiac enzyme levels, and ECG changes [34]. Subsequently, MI cases were diagnosed according to the criteria established by the European Society of Cardiology and the American College of Cardiology [35].

### 2.3. Non-Targeted Metabolite Profiling

Non-targeted metabolite profiling was conducted on the serum samples obtained from the participants of the baseline KORA S4 study using the Metabolon analytical system (Metabolon Inc., Durham, NC, USA). To ensure data quality, the same rigorous quality control criteria as described in detail in a previous study were applied [36]. Briefly, in the dataset of KORA S4, metabolites with more than 20% missing values were excluded; in addition, participants with levels of missing metabolites over 10% were similarly excluded. In total, we obtained a comprehensive panel of 363 metabolites from the KORA S4 study (for a complete list, refer to Supplementary Table S1 in Adam et al.) [36]. To prepare the data for analysis, all normalized relative ion counts were log-transformed due to the wide range of variation and a right-skewed distribution, and the remaining data were imputed with Multivariate Imputation by Chained Equations (MICE) [37].

To facilitate meaningful comparisons and eliminate the influence of non-fasting participants, each metabolite was standardized using min-max normalization. Min-max normalization, also known as feature scaling, is a commonly used data preprocessing technique that rescales numerical features to a specific range. In our study, all feature values were transformed into a new range, typically between 0 and 1, based on the minimum and maximum values of each feature. This normalization method effectively handles the presence of extreme outliers and ensures that all features are on a comparable scale for further analysis.

### 2.4. Study Participants and Clinical Variables

The current analysis includes only KORA S4 participants who have undergone non-targeted metabolite measurements. Individuals who had a history of MI before or during the baseline S4 phase, as well as those without participation of the F4 study, were excluded from the analysis. After the data cleaning process, the final dataset consists of 78 incident MI cases and 1376 non-MI individuals.

Demographic and clinical variables, including personal interviews, anthropometric measurements, and laboratory measurements of blood samples, were collected during the KORA S4 study and have been described elsewhere [38,39]. For instance, participants were stratified into three glucose tolerance groups according to the WHO diagnostic guidelines, based on fasting glucose levels and 2 h glucose values following an oral 75 g glucose load: (1) normal glucose, individuals with fasting glucose levels below 110 mg/dL and 2 h glucose values under 140 mg/dL; (2) prediabetes, which includes individuals with impaired fasting glucose (fasting glucose levels between 110 and 125 mg/dL), and/or impaired glucose tolerance (2 h glucose readings between 140 and 199 mg/dL); and (3) type 2 diabetes, which encompasses both newly diagnosed individuals and those with a known history of diabetes, as confirmed by physician-validated self-reporting. Diagnostic criteria for this group are fasting glucose levels of 126 mg/dL or higher, and/or 2 h glucose values of 200 mg/dL or above.

In the current study, a total of 19 clinical variables were analyzed, and the baseline characteristics of the study participants are presented in Table 1. Significant differences (*p*-values < 0.001) were observed between the incident MI and non-MI groups in sex, waist-to-hip ratio, fasting glucose, glucose tolerance groups, and high-sensitivity C-reactive protein (hs-CRP) levels. The incident MI group consisted predominantly of males but exhibited lower fasting glucose levels and lower hs-CRP levels compared to the non-MI individuals.

### 2.5. Development of the GAN Model for Feature Enhancement

The observational dataset used in our study comprises 1454 individuals, each with 382 features, including 19 clinical variables and 363 metabolites. However, the number of incident MI cases is limited, and there is a significant imbalance between the number of incident MI participants and the number of non-MI participants in the dataset. This imbalance could potentially impact the specificity and sensitivity of our models. To address this issue, we employed the k-NN clustering algorithm to divide the data into three groups.

Each group was subsequently divided into training, testing, and validation datasets using a 64%/16%/20% split, respectively. This division process was performed to ensure that the data from each group were representative and to evaluate the performance of our models effectively. Finally, the divided data from each group were merged to form the final dataset.

**Figure 1:**
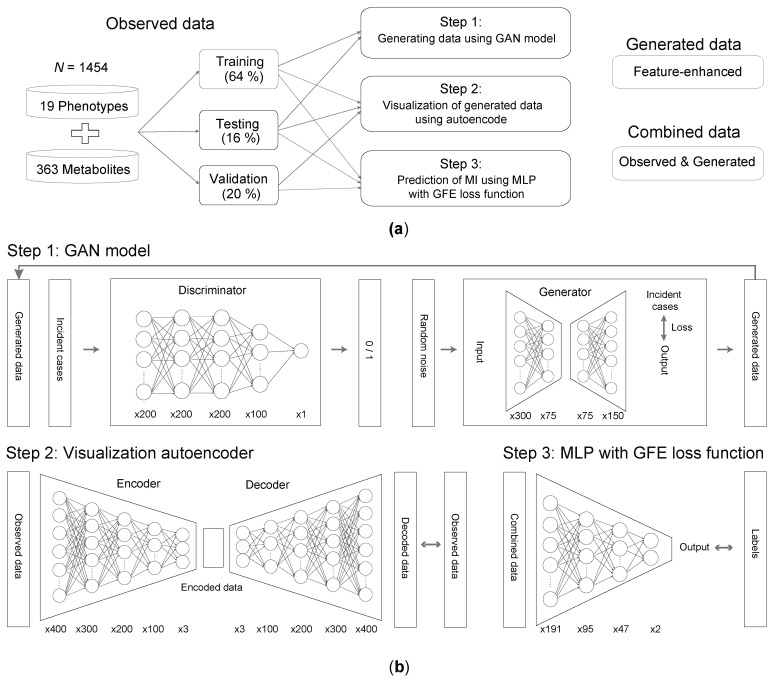
Roadmap and structure of deep-learning models. (**a**) Study design outlining the three steps used to train the prediction model, along with the datasets involved at each stage. (**b**) Overview of the DL model construction in three steps. The number below represents the number of neurons in each layer. Step 1: GAN model construction, which comprises a discriminator and a generator. The discriminator begins with an input layer of 382 features, followed by three hidden layers with 200 neurons each, designed to progressively capture patterns. An intermediate layer followed by one subsequent layer with 100 neurons allows the network to refine its learned features. The output layer culminates in a single neuron that outputs the prediction through a sigmoid activation function. For the generator, we employed an autoencoder model. The generator consists of an encoder and a decoder. In the encoder, Layer 1 compresses the input to 300 neurons, followed by BatchNorm and Rectified Linear Unit (ReLU) processing. Layer 2 further compresses to 75 neurons, again followed by BatchNorm and ReLU processing. The decoder’s Layer 1 expands from 75 to 150 neurons, and uses BatchNorm and ReLU. Layer 2 restores the original dimensionality with 382 features. Each hidden layer is equipped with ReLU activation to introduce non-linearity, aiding in learning more complex functions. Batch normalization is applied after each ReLU activation to stabilize learning and improve convergence rates. We employed the Adam optimizer for training our models, with the learning rate set at 0.005 and weight decay at 0.0001. The models were trained for 3000 steps to ensure convergence. In Step 2, for visualizing the generated data, we designed another autoencoder. The encoder’s input layer accepts an input of 382 features, which it further compresses through five layers (400, 300, 200, 100, and then down to 3 neurons), each followed by a LeakyReLU activation function. The decoder’s Layer 1 expands from the bottleneck of 3 neurons to four additional layers (100, 200, 300, and finally 400 neurons). We employed the Adam optimizer for training this model, setting the learning rate set at 0.0001. The models were trained for 2000 epochs to ensure effective learning and optimal convergence. In Step 3, we designed an MLP model for the final prediction. The model starts with an input of 382 features, which it sequentially reduces to half its size (191 neurons), then to a quarter of the original input size (95 neurons), and then to one-eighth of the original input size (47 neurons). The MLP model was also trained using the Adam optimizer, setting the learning rate at 0.00005 and weight decay at 0.0001, and then training for 200 epochs to ensure thorough learning and convergence of the model.

Given the limited number of incident MI cases in the observational data, training DL models directly on this data can be challenging. Therefore, we adopted a two-step approach, starting with training a GAN model to generate synthetic incident MI cases (Step 1 in Figure 1a,b).

The GAN model consists of two complementary components: a generator (G) and a discriminator (D). The generator is responsible for learning the patterns of incident MI cases and generating new data, while the discriminator is trained to distinguish between real observed data and data generated by the GAN model. The generator and discriminator are trained concurrently, with the goal of increasing the prediction accuracy of the discriminator, which indicates that the generated data has become more similar to the real observational data [40]. Subsequently, the training and test datasets are fed into the GAN model, and the generated data is referred to as “feature-enhanced” data. In this study, the feature-enhanced data consists of 2600 individuals, encompassing the 19 clinical variables and 363 metabolites.

### 2.6. Construction of the GAN for Feature-Enhanced Loss Function

To address the potential presence of misleading information in the generated data that could deviate the gradient from the ground truth optimal, we have developed a novel loss function called GAN for feature-enhanced (GFE). The objective of the GFE loss function is to reduce the impact of misleading information and bring the lowest point of gradient descent closer to the ground truth. This is accomplished by calculating the actual training direction while emphasizing the original observed incident case data.

The GFE loss function, as shown in Equation (1), is an extension of the binary cross entropy (BCE) loss function, designed to further enhance prediction accuracy. In the equation, we extract the portions of misleading information from the generated data during each training epoch, which refers to one complete pass of the entire training dataset through the learning algorithm. The discriminator (D) used in the GAN model is a previously fully trained model. To evaluate the reliability of the generated data, we utilize the accuracy of D(P), which represents the discriminator’s accuracy when evaluating the generated data. The term (1-Acc(D(P))) quantifies the misleading information contained in the generated data.

It is important to note that the discriminator is trained on incident cases and has learned information during the training process. Therefore, we also evaluate the reliability of the discriminator. Since non-MI cases are not used in any phase of the GAN training process, we use Acc(D(N)) as an appropriate evaluation method for the discriminator.

The term BCE(X’) in the GFE loss function emphasizes the ground truth information for gradient descent. X represents the combined data, which includes both observed data and generated data. X’ specifically refers to the observational positive data, which is used to generate the misleading information in the generated data. The weight W represents the weight of the observed portion in the batch size divided by the total training number.

GFE () = BCE(X) + W × (1-Acc(D(P)) × Acc(D(N)) × BCE(X’)(1)

where:

GEF () = GAN for feature enhanced loss function;

BCE () = binary cross entropy loss function;

X = combined data = observed data + generated data;

X’ = observational positive data;

W = weight of observed portion in BatchSize/Total training number;

Acc(D(P)) = accuracy of Discriminator (Observational positive data);

Acc(D(N)) = accuracy of Discriminator (Observational negative data).

By incorporating the GFE loss function, we emphasize the ground truth direction by utilizing purely observational positive data. Simultaneously, we account for and mitigate the effect of misleading information contained in the generated data during each training epoch.

### 2.7. Using the Autoencoder Model to Exhibit the Observed and Generated Data

To evaluate the generative quality of the GAN model and better understand the distribution relationship between the feature-enhanced data and the observed data, we employ an autoencoder model. The autoencoder is utilized to embed both the observed and generated data into three representative features, allowing us to visualize and compare the distributions of these data points in a three-dimensional coordinate system [41] (Step 2 in Figure 1a,b).

An autoencoder is a type of artificial neural network that is trained to reconstruct its input data. It consists of two main components: an encoder and a decoder. The encoder compresses the input data into a lower-dimensional representation, while the decoder aims to reconstruct the original input from this compressed representation. By training the autoencoder on the observed and generated data, we can learn meaningful features that capture the underlying structure of the data.

In our study, we train the autoencoder model using the combined observed and generated data. The autoencoder is specifically designed to produce a three-dimensional representation of the data. This allows us to visualize the distribution of the observed and generated data points in a three-dimensional coordinate system, which provides insights into the generative quality of the GAN model.

By comparing the distributions of the observed and generated data in this three-dimensional space, we can assess how well the GAN model captures the underlying distribution of the observed data. This analysis helps us understand the similarities and differences between the observed and generated data points, providing valuable insights into the performance and effectiveness of the GAN model in generating realistic data.

### 2.8. Using SMOTE + ENN and MLP to Improve Prediction of MI

After combining the newly generated feature-enhanced data with the observational training data, we employ a technique called synthetic minority oversampling technique (SMOTE) in conjunction with edited nearest neighbor (ENN) to improve the prediction of MI [42]. This approach helps us avoid both the overfitting and the underfitting issues that can arise when dealing with imbalanced datasets.

The combined dataset, consisting of the generated data and the observational training data, is incrementally augmented using SMOTE + ENN sampling in batches of 100. After each batch addition, the SMOTE + ENN technique is applied, which oversamples the minority class (MI cases) using synthetic examples generated by interpolating between neighboring instances, and then removes noisy and borderline instances using the ENN algorithm. The iterative sampling process continues until the count of non-MI individuals approaches 1000, indicating a well-balanced and comprehensively characterized dataset. This ensures that we have a sufficient representation of both the MI and non-MI cases in the dataset, reducing the bias towards the majority class.

Once the dataset is appropriately balanced, we construct a multi-layer perceptron (MLP), a type of deep neural network model, to predict incident and non-MI cases (Step 3 in Figure 1a,b) [43]. The MLP model is trained using the augmented dataset, which includes the generated data and the observational training data. After training the MLP model, we evaluate its performance by assessing accuracy and sensitivity using separate test and validation datasets. Accuracy measures how well the model predicts both incident and non-MI cases, while sensitivity focuses specifically on the model’s ability to correctly identify MI cases.

To compare the performance of the GAN model with the MLP model we introduced, we also utilize other ML models such as DT, RF, and SVM. Additionally, we explore DL models including convolutional neural networks (CNN) and long short-term memory networks (LSTM). These models are trained and evaluated using the same observational dataset to predict incident MI cases [44,45].

By comparing the performance of different ML and DL models, including the GAN-based MLP model, we can gain insights into their respective strengths and weaknesses in the prediction of myocardial infarction.

### 2.9. Sensitivity Analyses

After training the model, we conducted additional interpretation to identify potential patterns within the data and gain insights into the important clinical variables or metabolites for the prediction of MI generated by the model.

To perform sensitivity analyses, we focused on understanding the impacts of individual clinical variables or metabolites on the model’s predictions. Firstly, we calculated the mean value of each phenotype or metabolite separately for the incident MI cases and non-MI participants. This allowed us to compare the average values between these two groups and identify potential differences. Next, we replaced each incident MI case with the mean value of the corresponding non-MI individual for a specific phenotype or metabolite. This replacement was performed one variable at a time, and the modified data were then fed into the fully trained model for prediction. We repeated this process for each clinical variable or metabolite of interest.

To quantify the extent of change, we documented the model’s output variance before and after each incident MI case was replaced with the mean value from the non-MI individuals. By comparing the variances, we could assess how much the model’s predictions were influenced by the replacement of the original data points with mean values. This sensitivity analysis procedure was executed on each dataset, and we computed the average change values for every phenotype or metabolite across all datasets. These average change values served as a measure of how influential each predictive variable was within the model. They reflected the individual contribution of each data point to the model’s predictive power. By analyzing the average change values, we gained insights into the importance of different clinical variables and metabolites in the model’s predictions of MI. This information allowed us to identify the most influential factors and understand their impacts on the overall predictive performance of the model.

### 2.10. Language Enhancement Method 

We employed ChatGPT to enhance the linguistic quality of this manuscript, specifically focusing on the improvement of English grammar. Additionally, we have thoroughly reviewed all modifications suggested by ChatGPT to ensure that the original context and scientific integrity of the paper were preserved.

## 3. Results

### 3.1. Visualization of Feature-Enhanced Data Using the Autoencoder Model

**Figure 2:**
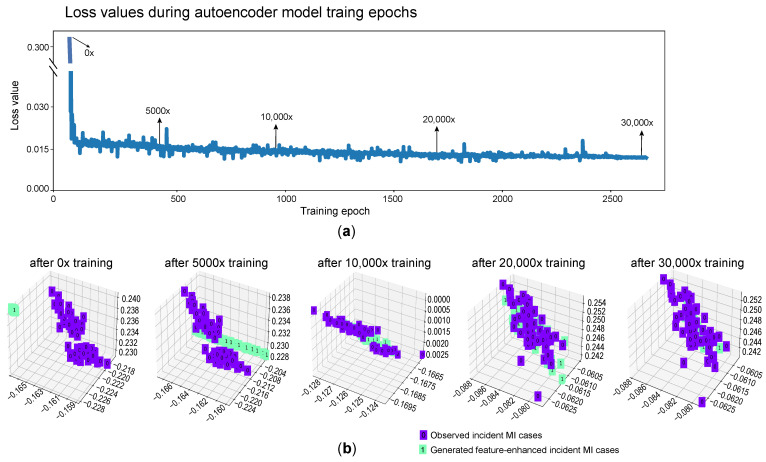
Autoencoder training results. (**a**) This plot demonstrates how the loss values changed during the model training epoch. As the training progressed, the loss value gradually decreased and eventually stabilized at a minimal level. This indicates that the model was fully trained and able to capture the important features of the data. (**b**) This plot illustrates the changes in the differences between the generated data and the observed incident MI cases resulting from the GAN model with various training times. The green nodes represent the generated data, while the purple nodes represent the observed incident MI cases. It can be observed that, as the training time increased, the generated data became more aligned with the observed incident MI cases, indicating the improvement in the model’s ability to generate data that closely resembles the real cases.

To gain insights into the feature-enhanced data produced by the autoencoder model, we conducted a comparative analysis. As the training period of the autoencoder model increased, we observed a continuous decrease in the loss value, indicating that the model’s parameters were being iteratively adjusted to achieve optimal results (Figure 2a). After 2500 training epochs, the loss values began to stabilize, suggesting that the model had reached a satisfactory level of training.

Subsequently, we utilized the encoder model to compress both the generated and observed data, resulting in a low-dimensional representation for each data point. The encoder reduced the data to three numerical points, which served as the most representative embeddings. To visualize the distribution of the feature-enhanced data, we plotted the generated and observed incident MI cases in a three-dimensional coordinate system (Figure 2b).

Initially, the distribution of the generated data, produced at the beginning of the model training process (e.g., without and after 5000 training iterations), differed from the distribution of the observed data. However, as the training time increased (e.g., after 20,000 training iterations), the distribution of the generated data gradually aligned with the distribution of the incident MI cases. Remarkably, after training with 30,000 iterations, the generated data distribution closely matched that of the incident cases (Figure 2b).

It is important to note that this alignment in distribution does not imply that the generated data and the observed incident MI cases were identical. Instead, it signifies that they exhibited similarity in terms of the feature distribution within the low-dimensional space. In the original high-dimensional space, the generated data remained distinct from the observed incident MI cases.

The visualization of the feature-enhanced data through the autoencoder model provided valuable insights into the progression of the model training and its ability to capture the distribution patterns of the incident MI cases. It demonstrated the convergence of the generated data distribution with the observed data distribution over time, highlighting the model’s capability to generate synthetic data that mimicked the feature distribution of the incident MI cases in the lower-dimensional space.

### 3.2. Characteristics of the Generated Dataset

**Figure 3:**
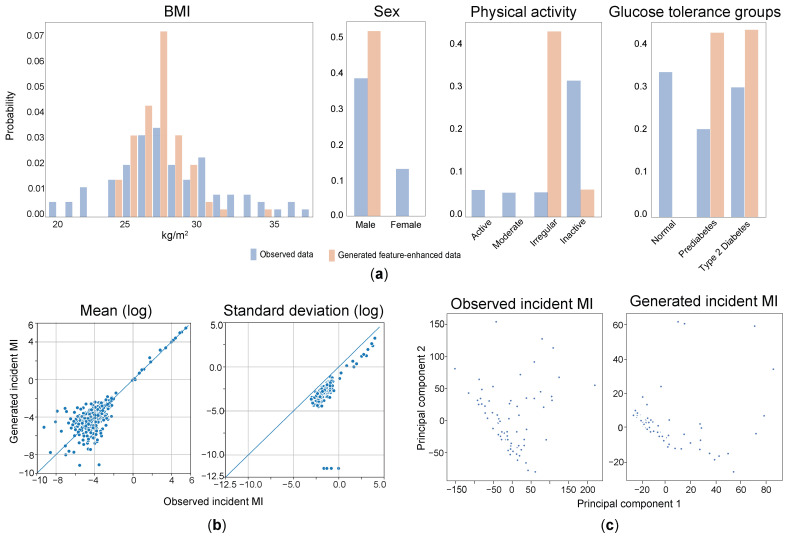
Comparison between feature-enhanced data and observed incident myocardial infarction cases. (**a**) Four plots illustrate the distributions of four clinical variables (BMI, sex, physical activity, and glucose tolerance groups) in the generated and observed datasets, respectively. The distribution of a subset of eigenvalues for each variable is shown. We observe a normal distribution trend in the generated data that closely resembles the observational data for BMI, but male sex and irregular and inactive physical activity, as well as prediabetes and type 2 diabetes in glucose tolerance groups, are mainly present in the generated dataset. (**b**) Here, we present the scatter plots showing the correlations between the generated and observed data. The points on the scatter plot are close to the diagonal line y = x, indicating that the mean and standard deviation of the generated data are similar to those of the observed data. (**c**) After-dimensional reduction using PCA (Principal Component Analysis); the trend of the feature-enhanced data is closer to the incident cases. We selected the most significant features for enhancement, and the results of the PCA analysis show that the feature-enhanced data aligns more closely with the observed incident MI cases.

The characteristics of the generated feature-enhanced data were evaluated by comparing them with the observed incident MI cases. During the training process, the generator competed with the discriminators and optimized itself. As a result, the generator selected variables with high confidence to deceive the discriminators, indicating significant differences in those variables’ features. For example, in the observational dataset of incident MI cases, the proportion of females was only 26% (Table 1). To generate more reliable data, the generator focused on generating characteristics primarily associated with males, such as male sex and other similar attributes like irregular physical activity, prediabetes, and type 2 diabetes (Figure 3a). The exclusion of data from females in the generated dataset led to changes in correlation and standard deviation when comparing the observed and feature-enhanced data (Figure 3b). The absence of data relating to female subjects resulted in altered relationships and variability between variables in the generated dataset compared to the observed dataset.

These findings highlight that the generator, through the competitive optimization process, prioritized variables that exhibited significant feature differences and had a higher potential to deceive the discriminators. As a consequence, the generated feature-enhanced data may not fully represent the characteristics of the observed incident MI cases, especially in terms of variables related to female sex and other associated attributes.

### 3.3. Improvement of Prediction Accuracy Using the MLP Model

**Figure 4:**
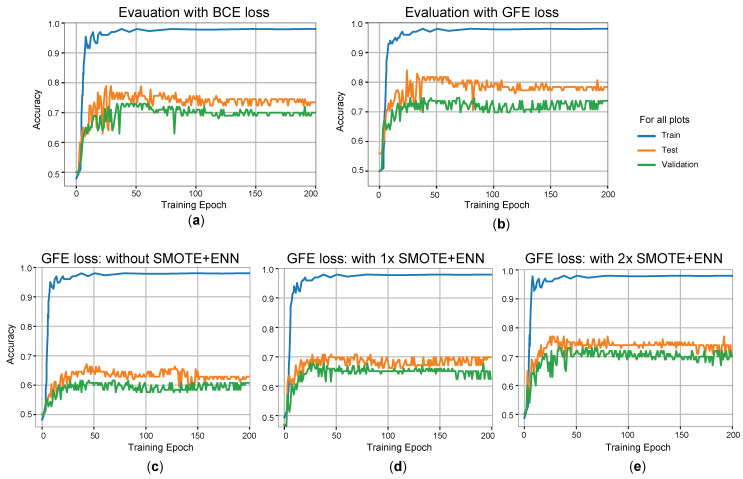
The loss curves of our models in various conditions as depicted by the MLP results. We replicated the entire process by splitting the combined dataset into training, testing, and validation datasets ten times, and each time, we replicated the prediction model training process and evaluated the model using test and validation datasets. (**a**) MLP training results with BCE loss. (**b**) MLP training results with GFE loss. (**c**–**e**) The variation in ACC in the MLP training process under different situations. (**c**) The training curve obtained directly from the combined data. (**d**) The training process curve obtained after a one-time SMOTE+ENN sampling; the accuracy rate of the validation dataset occasionally varied from 64% to 68%, resulting in unstable training results. (**e**) Two SMOTE+ENN sampling processes were conducted, and the training results were more stable. The accuracy of the prediction of MI, compared with the validation dataset, was approximately 70%.

To improve the prediction accuracy, we utilized the MLP model with the combined feature-enhanced and observed dataset. Initially, we compared the conventional BCE loss function (Figure 4a) with our proposed GFE loss function (Figure 4b). The model achieved its highest performance on the test dataset after 50 training epochs. In Figure 4a, as the training time increased, the model’s prediction accuracy approached 100% on the training dataset, 74% on the test dataset, and 70% on the validation dataset, which were averaged over ten training runs. However, as the model’s error information was based on the generated data, its actual prediction performance degraded with further training due to a biased gradient descent direction.

By using our proposed GFE loss function instead of the conventional BCE loss function, we observed a 2% improvement in the model’s actual prediction result under the same training conditions (Figure 4b). However, the model inevitably learned incorrect information from the generated data. The prediction accuracy of the model on the test dataset remained at 78%, while it was 74% for the validation dataset. As the test dataset was used during GAN training, the generated data contained some information from the test dataset, resulting in a slightly higher prediction accuracy on the test dataset compared to the validation dataset. We propose that the model’s performance on the validation dataset represents its real prediction accuracy, as the validation dataset is entirely independent.

Using the GFE loss function and the combined dataset, we further evaluated the MLP model without SMOTE+ENN (Figure 4c), with one-time SMOTE+ENN (Figure 4d), and with two-times SMOTE+ENN (Figure 4e). The prediction accuracy of the model on the validation datasets improved, with the highest accuracy achieved using two-times SMOTE+ENN. The MLP model achieved prediction accuracies of approximately 60%, 65%, and 70% for the validation dataset without SMOTE+ENN, with one-time SMOTE+ENN, and with two-times SMOTE+ENN, respectively (Figure 4c–e).

**Figure 5:**
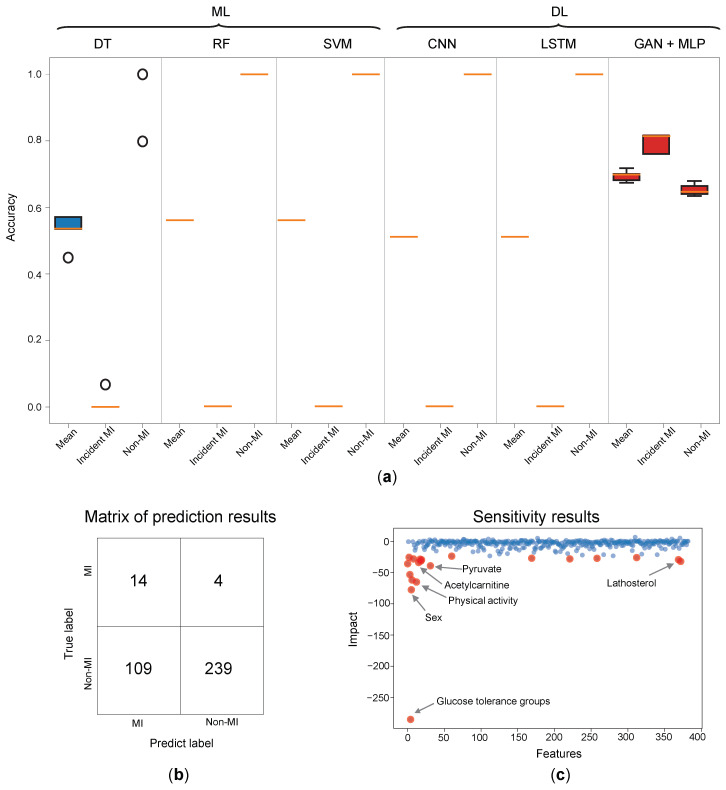
(**a**) The comparison between myocardial infarction prediction models (KORA Study). Due to the disparity between incident MI cases and non-MI individuals in the test and validation datasets, the model’s accuracy is determined by averaging the accuracy values for incident MI and non-MI cases of 50 outcomes. It compares our methods to five common methods, both ML (SVM, RF, and DT) and DL (CNN and LSTM). In addition, each method displays the model’s accuracy in predicting incident cases and non-MI cases, as well as the overall average accuracy on the validation dataset. The orange lines indicate that the prediction results were the same and overlapped despite being trained at different times. (**b**) Confusion metrics of the prediction model’s performance on the validation dataset. (**c**) Illustration of the significance of numerous model variables, including phenotypes and metabolites. When the change in sensitivity was larger, which indicated that the variable was more significant, the change in the model’s prediction values increased. The blue dots correspond to all variables, while the red dots refer to the top 20 variables with the most significant changes in sensitivity.

In addition to our proposed DL (GAN with MLP) model, we constructed conventional ML models (DT, RF, and SVM) and DL models (CNN and LSTM) using the observed dataset to compare and assess the approaches. Due to the imbalanced data, with a large quantity of non-MI participants and limited quantity of incident MI cases, these models struggled to effectively learn from the incident cases. Consequently, to achieve the highest prediction accuracy, these models predicted all data points as non-MI, resulting in 100% accuracy for non-MI cases and 0% accuracy for incident MI cases with DT, RF, SVM, CNN, and LSTM approaches (Figure 5a).

The strategy we developed using the GAN with MLP models not only addressed the drawbacks of asymmetrical sampling but also enhanced key characteristics that contribute to the model’s effectiveness in data prediction, resulting in a 70% accuracy rate (Figure 4e). Finally, the confusion matrix results for this model are displayed in Figure 5b. The confusion matrix was constructed using the validation dataset, and the model achieved a prediction accuracy of 70%.

### 3.4. Sensitivity Analysis Results

Figure 5c presents the results of our model’s sensitivity analysis. We observed that the phenotypic data had greater impact weights on the model compared to the metabolite data. This finding suggests that the model’s prediction is more dependent on the clinical variables, with categories such as glucose tolerance, sex, and physical activity exhibiting the highest weights. Among the metabolites, acetylcarnitine, pyruvate, and lathosterol emerged as the top contributors to the model’s predictions, indicating their significant influence despite the lower average impact weight of metabolite data. While the average impact weight of metabolite data is lower than that of phenotypic data, the diverse distributions of metabolite data can enable the model to make more accurate predictions.

## 4. Discussion

In this study, we introduced a novel GFE loss function in our deep-learning algorithms, specifically in the GAN and MLP models. The incorporation of the GFE loss function resulted in a 2% improvement in the accuracy of our 7-year myocardial infarction (MI) predictions. Our approach involved three interconnected steps to achieve this improvement. Firstly, we utilized the GAN model to generate new incident MI cases based on real observational data obtained from the population-based KORA cohort. This approach allowed us to augment the dataset and strengthen the feature representation, enhancing the predictive capabilities of the models. Secondly, we implemented the MLP model and employed a combination of oversampling techniques, including SMOTE, and undersampling techniques, such as ENN. This approach aimed to address the issue of imbalanced data by balancing the combined generated feature-enhanced data with the real observational data. Finally, we introduced the GFE loss function, which is an adaptation of the conventional BCE loss function. By incorporating additional features and information from the dataset, the GFE loss function enhanced the accuracy of MI prediction. This improvement was achieved by leveraging the discriminative power of the GAN model and the predictive capabilities of the MLP model. The successful implementation of the GFE loss function, in conjunction with the GAN and MLP models, demonstrates its efficacy in improving the accurate prediction of MI. The findings highlight the potential of our approach in identifying novel biomarkers and enhancing risk stratification models for MI prediction.

The availability of high-quality and comprehensive clinical and metabolomic data is crucial to ensure accurate prediction of MI. In this study, we leveraged data from the KORA S4 and F4 studies, which provided a valuable resource for our analysis. To ensure data integrity, we implemented a rigorous data cleaning process for both clinical variables and non-targeted metabolite profiles. Specifically, we focused on utilizing incident MI cases and non-MI participants with measured metabolite data. By selecting only these individuals, we aimed to establish a more accurate representation of the population under study. However, it is important to acknowledge that this selection process introduced certain limitations to our dataset. One limitation was the relatively small number of observational MI cases available, which affected the overall balance of the data. The class imbalance, with a majority of non-MI cases, posed a challenge in training the models effectively. Imbalanced data can lead to biased predictions and inadequate performance, particularly when the minority class (MI cases) is of interest. As demonstrated in Figure 5a, when we initially applied ML methods such as DT, RF, SVM, and DL methods such as CNN and LSTM, the prediction of MI was highly misleading. These models achieved 100% accuracy for non-MI cases but failed to accurately predict any incident MI cases.

The limitations in our observational data, especially regarding the limited number of MI cases and the class imbalance, contributed to the challenges faced by the ML and DL models. These limitations highlight the importance of comprehensive and balanced datasets, including a sufficient number of MI cases, to improve the accuracy and reliability of predictive models. Addressing these limitations and ensuring the availability of complete and diverse data, particularly regarding clinical variables and metabolites, is essential to enhance the predictive accuracy of the models. An additional limitation of our model is that it is optimized solely for binary outcomes and does not extend to multinomial settings. Future studies should focus on expanding the dataset and incorporating more relevant variables to enhance the robustness and generalizability of MI prediction models.

To overcome the limitations arising from the limited number of incident cases, we employed a GAN model to learn from the observational data and generate new incident MI cases. However, even with the addition of the generated feature-enhanced data, the combined dataset remained largely imbalanced. To address this issue, we employed oversampling and undersampling techniques to balance the distribution space within the training dataset. Specifically, we utilized the SMOTE to generate synthetic samples, thereby increasing the representation of the minority class (incident MI). We then applied the ENN technique to remove noisy instances and further refine the dataset. This balanced dataset enabled the MLP model to effectively learn from the data and improve its performance on the minority class.

While we recognized the limitations of the generated data, we proposed a more appropriate loss function, the GFE loss function, which resulted in a 2% increase in prediction accuracy. The combination of the GAN model and the GFE loss function expanded the potential for generalization across different datasets. Dealing with imbalanced datasets, where the number of observed incident MI cases is significantly lower than that of non-MI individuals, presents challenges. Techniques like SMOTE and ENN help address this issue, but their effectiveness can vary depending on the dataset’s specific characteristics. In our study, through two rounds of training with SMOTE+ENN and the GFE loss function, we successfully employed deep-learning algorithms for the first time to predict 7-year incident MI cases in the KORA cohort data, achieving an accuracy of 70%.

However, our study also has additional limitations and potential challenges. The findings may be specific to the population and dataset used in the analysis, such as the KORA cohort. It is crucial to validate the performance of the deep-learning methods with the GFE loss function on external datasets to ensure their robustness and generalizability. Without proper validation on independent datasets, the reliability of the model’s predictions may be limited.

Deep-learning models, including generative adversarial networks, are often considered black boxes, making it challenging to interpret the underlying mechanisms and understand how the model arrives at its predictions. However, our sensitivity analyses ranked the impact weights of each clinical phenotype and metabolite in predicting MI, which may aid in the clinical understanding and acceptance of the model’s predictions. Our approach also established a novel method for enhancing the interpretability of the model, revealing numerous clinical variables and metabolites that are significantly relevant to MI. The identified clinical variables, such as the glucose tolerance groups, sex, and physical activity [46,47], align with those already recognized in current clinical tests, further demonstrating the reliability of our model. We anticipate that as generative deep-learning continues to advance, more cutting-edge techniques will emerge in the future to better target critical features and generate omics data, further improving the performance of predictive models [48,49,50,51,52].

## Figures and Tables

**Table 1:** Characteristics of the baseline KORA S4 study participants.

Clinical Variables	Incident MI *N* = 78	Non-MI *N* = 1376	*p*-Values
Age, year	65.50 [62.00, 70.00]	64.00 [59.00, 68.00]	0.005
Female sex, %	25.61	52.07	<0.001
BMI, kg/m^2^	28.72 [26.79, 32.58]	27.93 [25.63, 30.90]	0.005
Waist-to-hip ratio	0.95 [0.90, 0.99]	0.90 [0.83, 0.96]	<0.001
Systolic BP, mmHg	144.75 [128.62, 159.62]	135.00 [122.00, 148.00]	0.001
Diastolic BP, mmHg	80.00 [73.50, 87.00]	82.25 [74.12, 89.00]	0.100
Total cholesterol, mg/dL	237.10 [215.10, 263.15]	242.40 [214.57, 269.60]	0.495
HDL cholesterol, mg/dL	52.80 [42.38, 59.58]	56.50 [46.40, 67.82]	0.003
LDL cholesterol, mg/dL	152.10 [126.27, 178.00]	153.65 [130.85, 181.93]	0.528
HbA1c (%)	5.60 [5.40, 5.90]	5.75 [5.50, 6.40]	0.001
Fasting glucose, mg/dL	99.00 [93.00, 109.00]	107.00 [97.25, 136.00]	<0.001
Alcohol intake, g/day	6.60 [0.00, 22.86]	7.93 [0.00, 20.00]	0.976
Smoker, %	15.39	13.72	0.321
Physical activity, %			0.001
Active (>2 h/week)	12.84	17.41	
Moderate (>1 h/week)	11.51	25.81	
Irregular (<1 h/week)	11.52	15.33	
Inactive	64.13	41.46	
Glucose tolerance groups, %			<0.001
Normal glucose	39.21	61.30	
Prediabetes	24.27	23.92	
Type 2 Diabetes	35.12	14.59	
Fasting, %	88.71	79.53	0.022
Stroke, %	2.20	3.81	0.568
Statin user, %	9.29	7.72	0.782
hs-CRP, mg/L	1.67 [0.83, 3.38]	2.55 [1.30, 7.00]	<0.001

Abbreviations: BMI, body mass index; BP, blood pressure; HDL, high-density lipoprotein; LDL, low-density lipoprotein; hs-CRP, high-sensitivity C-reactive protein; MI, myocardial infarction.

## Data Availability

The KORA data are governed by the General Data Protection Regulation (GDPR) and national data protection laws, with additional restrictions imposed by the Ethics Committee of the Bavarian Chamber of Physicians to ensure data privacy of the study participants. Therefore, the data cannot be made freely available in a public repository. However, researchers with a legitimate interest in accessing the data may submit a request through an individual project agreement with KORA via the online portal (https://www.helmholtz-munich.de/en/epi/cohort/kora). All codes are available at https://github.com/ShawnYu1996/GAN-for-MI.
